# 培美曲塞鞘内化疗在*EGFR*阳性非小细胞肺癌软脑膜转移患者中的疗效与安全性评估

**DOI:** 10.3779/j.issn.1009-3419.2025.106.23

**Published:** 2025-08-20

**Authors:** Tianli ZHANG, Xin CHEN, Cheng JIANG, Yongjuan LIN, Yu XIE, Huiying LI, Zhenyu YIN, Tingting YU

**Affiliations:** 210008 南京，南京大学医学院附属鼓楼医院老年肿瘤科; Department of Geriatric Oncology, Affiliated Nanjing Drum Tower Hospital of Nanjing University Medical School, Nanjing 210008, China

**Keywords:** 肺肿瘤, 软脑膜转移, 表皮生长因子受体, 鞘内注射化疗, 培美曲塞, Lung neoplasms, Leptomeningeal metastasis, Epidermal growth factor receptor, Intrathecal chemotherapy, Pemetrexed

## Abstract

**背景与目的:**

软脑膜转移（leptomeningeal metastasis, LM）在晚期非小细胞肺癌（non-small cell lung cancer, NSCLC）患者中的发病率逐年上升，但其治疗手段有限，预后较差。鞘内注射培美曲塞（intrathecal Pemetrexed, IP）在LM治疗领域具有广泛的应用前景。本研究旨在评估IP在表皮生长因子受体（epidermal growth factor receptor, *EGFR*）突变阳性NSCLC-LM患者中的疗效、安全性及最佳联合应用模式，以期为此类患者探寻更精准的个体化治疗策略提供真实世界数据支持。

**方法:**

回顾性收集2018年1月至2024年6月在南京大学医学院附属鼓楼医院接受IP治疗的104例*EGFR*突变阳性NSCLC-LM患者的临床资料、治疗方案和生存数据。系统分析入组患者的总生存期（overall survival, OS）、无进展生存期（progression-free survival, PFS）、临床有效率和不良反应（adverse events, AEs）。

**结果:**

104例*EGFR*突变阳性NSCLC-LM患者的中位PFS为9.6个月，中位OS为13.0个月，6个月和1年OS率分别为80.8%和56.5%，临床有效率为77.9%。常见的AEs是骨髓抑制（58.7%）和转氨酶升高（25.0%）。9例（8.7%）患者出现4级骨髓抑制，给予对症治疗后恢复正常。亚组分析显示，卡氏体能状态（Karnofsky performance status, KPS）评分≥60分的患者OS显著长于KPS<60分患者（14.4 *vs* 9.0个月，*P*=0.0022）。规律接受贝伐珠单抗治疗的LM患者中位OS明显高于未接受贝伐珠单抗治疗者（19.2 *vs* 10.5个月，*P*=0.0011）。

**结论:**

IP治疗在*EGFR*突变阳性NSCLC-LM患者中具有良好的疗效和安全性，联合贝伐珠单抗可发挥协同抗肿瘤作用，进一步改善预后。

## Abstract (en)

**Background and objective:**

The incidence of leptomeningeal metastasis (LM) in patients with advanced non-small cell lung cancer (NSCLC) is increasing gradually. However, it poses therapeutic challenges due to limited effective interventions. Intrathecal Pemetrexed (IP) holds broad application prospects in the therapeutic domain of LM. This study aims to evaluate the efficacy, safety, and optimal combination strategies of IP in NSCLC-LM patients with epidermal growth factor receptor (*EGFR*) mutation-positive status, with the aim of providing real-world data support for exploring more precise personalized treatment strategies for these patients.

**Methods:**

104 *EGFR*-mutated NSCLC-LM patients who received IP treatment at Affiliated Nanjing Drum Tower Hospital of Nanjing University Medical School from January 2018 to June 2024 were analyzed retrospectively. Clinical parameters, treatment regimens, and survival outcomes were collected. The overall survival (OS), progression-free survival (PFS), clinical response rate and adverse events (AEs) were evaluated.

**Results:**

The cohort demonstrated a median PFS of 9.6 months and OS of 13.0 months with 6-month and 1-year OS rates of 80.8% and 56.5%, respectively. Clinical response was observed in 77.9% of patients. The common AEs were myelosuppression (58.7%) and elevation of hepatic aminotransferases (25.0%). Nine (8.7%) patients experienced grade 4 myelosuppression and recovered to normal after receiving symptomatic treatment. Subgroup analyses revealed prolonged OS in patients with Karnofsky performance status (KPS) ≥60 versus <60 (14.4 *vs* 9.0 months, *P*=0.0022) and those receiving Bevacizumab therapy versus not (19.2 *vs* 10.5 months, *P*=0.0011).

**Conclusion:**

IP exhibits promising efficacy and manageable toxicity in *EGFR*-mutated NSCLC-LM patients. When combined with Bevacizumab, it exerts synergistic antitumor effects with the potential to further improve clinical outcomes.

软脑膜转移（leptomeningeal metastasis, LM）是指恶性肿瘤细胞浸润软脑膜、蛛网膜下腔、脑脊液的一种中枢神经系统严重并发症，患者常伴随顽固的头痛、恶心、剧吐，以及多种颅高压、神经缺损的症状，生活质量极差^[1]^。在非小细胞肺癌（non-small cell lung cancer, NSCLC）中，LM的发生率为3%-5%，而在表皮生长因子受体（epidermal growth factor receptor, EGFR）突变阳性的患者中，其发生率可达9.4%^[2,3]^。针对NSCLC-LM目前尚无标准治疗指南或共识，常规治疗手段包括全身化疗、EGFR-酪氨酸激酶抑制剂（EGFR-tyrosine kinase inhibitors, EGFR-TKIs）治疗、免疫治疗、全脑放疗（whole brain radiation therapy, WBRT）等^[4-6]^。但由于血脑屏障（blood brain barrier, BBB）的限制，抗肿瘤药物很难进入中枢达到并维持有效的药物浓度，患者通常预后较差，中位生存期仅3个月左右^[7]^。目前，LM的治疗仍是肺癌领域的共识性难题，亟需深化疾病认知及研发新的诊疗策略。

近来，鞘内注射化疗因其可突破BBB直接将药物递送至脑室内，成为LM治疗突破的重要方向。传统鞘内注射化疗药物包括甲氨蝶呤、阿糖胞苷、塞替派等，在NSCLC-LM患者中疗效有限且副作用较大^[2,8]^。培美曲塞是NSCLC一线化疗药物，但全身用药时脑脊液中浓度低。前期动物实验及小样本的临床研究^[9,10]^初步证实了其鞘内化疗的有效性和安全性。但目前仍缺乏培美曲塞鞘内化疗（intrathecal Pemetrexed, IP）治疗*EGFR*突变阳性NSCLC-LM患者的大样本真实世界研究。因此，本研究系统地回顾了南京大学医学院附属鼓楼医院经治的此类患者的诊疗经过及预后情况，旨在探明“IP治疗*EGFR*突变阳性NSCLC-LM”这一治疗策略的有效性、安全性及最佳治疗模式等，初步建立该治疗方案完善的技术方法学、疗效评估体系和质量监控指标，为晚期难治性LM患者提供新的个体化精准治疗途径。

## 1 资料和方法

### 1.1 研究对象

回顾性分析2018年1月至2024年6月在南京大学医学院附属鼓楼医院就诊的*EGFR*突变NSCLC-LM患者。纳入标准：（1）年龄>18岁；（2）病理确诊为肺腺癌；（3）肺组织或外周血基因检测为*EGFR*突变；（4）脑脊液细胞学或头颅磁共振成像（magnetic resonance imaging, MRI）（脑膜强化或脑积水型）明确诊断为LM；（5）病程中至少接受2次IP治疗；（6）无严重肝肾功能障碍，白细胞计数≥3.0×10^9^/L，血小板计数≥100×10^9^/L。排除标准：（1）人类免疫缺陷病毒阳性；（2）严重感染或严重的合并病，如消化性溃疡出血性肠梗阻、心力衰竭、肾衰竭或控制不良的糖尿病；（3）对培美曲塞过敏；（4）患者或其家属拒绝接受侵入性操作。本研究获南京大学医学院附属鼓楼医院伦理委员会批准（批准号：2024-544-02）。

### 1.2 治疗方案

所有患者均接受规律的IP治疗。具体给药方法为：将培美曲塞200 mg冻干粉（0.2 g/瓶，批号：602250501，江苏豪森药业集团有限公司，30 ^o^C以下储藏保存，遮光密封，有效期18个月）溶解于10 mL 0.9%生理盐水中，随后取1-2.5 mL（20-50 mg培美曲塞）药液，通过腰椎穿刺或Ommaya囊进行给药。给药时间点为第1天和第8天，每4周为1个治疗周期，直至出现颅内疾病进展或不可接受的毒性反应等原因需要停止治疗。

在鞘内注射培美曲塞之前，患者需接受地塞米松5 mg鞘内注射，以预防可能的不良反应（adverse events, AEs）。每次鞘内注射化疗药物前，均需测量患者的脑脊液压力，并根据测量结果进行个体化的全身治疗和支持治疗，以确保患者的安全和治疗的有效性。此外，所有接受IP治疗的患者还需口服叶酸（400 μg，每日1次）和维生素B_12_（1000 μg，在第一剂培美曲塞前1-2周肌肉注射，随后每次治疗重复一次），以降低药物毒性，提高治疗耐受性。

关于IP治疗的最佳剂量和给药时间，目前尚未有统一的标准。剂量应根据患者脑脊液体积和药物代谢情况动态调整，根据本研究团队前期临床研究结果及相关文献^[10,11]^，确定初始剂量为20 mg，随后每次增加10 mg，直至最大剂量50 mg，当患者出现3级及以上AEs或不可耐受时，剂量恢复至之前的剂量水平。前期研究中IP给药模式多为诱导期每周2次，但在维持治疗中差异很大^[9,11,12]^。基于培美曲塞在脑脊液中的半衰期较长及患者耐受性，第1天和第8天每4周给药一次可维持有效治疗浓度，同时避免峰浓度过高引发的神经毒性。

患者接受IP治疗的注射过程中，根据患者情况制定个体化的全身治疗方案。所有经典*EGFR*突变患者均接受双倍剂量第三代EGFR*-*TKIs靶向治疗，部分患者每个IP周期进行一次贝伐珠单抗400 mg抗血管生成治疗，但严禁同时给予静脉化疗、放疗或免疫治疗，治疗持续至疾病进展或出现无法耐受的AEs。确诊LM后、鞘内化疗前进行过静脉化疗、放疗或免疫治疗的患者仍可被纳入研究。

### 1.3 疗效与安全性评估

基于神经肿瘤疗效评估标准（Response Assessment in Neuro-Oncology, RANO）进行临床有效性评估^[13]^。RANO标准包括神经系统检查、影像学评估、脑脊液细胞学。本研究临床有效性评估主要包括3个方面：（1）每周期IP前进行临床症状与功能状态评估，包括症状、头痛评分、卡氏体能状态（Karnofsky performance status, KPS）评分；（2）每2个月进行一次脑膜增强MRI检查；（3）每周期行IP治疗时留取脑脊液进行癌胚抗原（carcinoembryonic antigen, CEA）检测。无进展生存期（progression-free survival, PFS）定义为第一次鞘注至颅内或颅外进展或死亡的时间。总生存期（overall survival, OS）定义为第一次鞘注至研究终点（死亡、失访或随访截止日期）的时间。随访截止日期为2025年3月15日。AEs主要包括：骨髓抑制（白细胞减少、血小板减少）、转氨酶升高（elevation of hepatic aminotransferases, EHA）、癫痫发作、急性脑膜炎、脑白质病。通过不良事件通用术语标准（Common Terminology Criteria for Adverse Events, CTCAE）对AEs进行分级和记录。

### 1.4 统计分析

数据使用统计软件SPSS 27.0版本（IBM Corp., Chicago, IL, USA）进行分析。采用*Kaplan-Meier*法进行生存分析并绘制生存曲线，组间差异的比较使用*Log-rank*检验。使用多变量*Cox*比例风险模型评估亚组间交互作用，控制混杂因素。*P*<0.05为差异有统计学意义。

## 2 结果

### 2.1 临床特征

研究共纳入104例*EGFR*突变阳性NSCLC-LM患者，年龄35-75岁，中位年龄为56岁；男性和女性占比分别为47.1%和52.9%。诊断LM时65例（62.5%）患者KPS评分≥60分，39例（37.5%）患者KPS评分<60分。经典突变中，19外显子缺失突变占比为42.3%（44例），21外显子L858R突变占比为40.4%（42例）。罕见突变中，21外显子L861Q突变占比为1.9%（2例），18外显子G719A/G719S/G719X突变占比为3.8%（4例），20外显子插入占比为8.7%（9例）。68例（65.4%）患者确诊肺癌2年以上发生LM；确诊LM时，68例（65.4%）同时合并脑转移，73例（70.2%）同时合并颅外转移。92例（88.5%）患者根据脑脊液细胞学阳性诊断，12例（11.5%）脑脊液阴性患者依据症状和影像学确诊。确诊LM前，分别有66例（63.5%）、9例（8.6%）、16例（15.4%）患者接受第一代、二代、三代TKIs治疗。确诊LM后，95例（91.4%）患者接受了第三代TKIs治疗，其中经典突变患者均接受了双倍剂量第三代TKIs治疗。44例（42.3%）患者在IP治疗每周期均接受一次400 mg贝伐珠单抗静脉治疗，中位给药周期为6（范围：2-14）。26例（25%）患者确诊LM后接受过静脉化疗，中位周期数为4（范围：2-6）。6例（5.8%）患者接受过头颅放疗，均为合并脑实质转移患者。104例患者中有29例（27.9%）确诊后通过植入Ommaya囊经囊行IP治疗，其余患者经腰椎穿刺予培美曲塞鞘注。患者的详细临床特征见表1。

**表1:** NSCLC-LM患者的临床特征（*n*=104）

Variables	*n* (%)
Age (yr)	
≥60	28 (26.9)
<60	76 (73.1)
Gender	
Male	49 (47.1)
Female	55 (52.9)
KPS score	
≥60	65 (62.5)
<60	39 (37.5)
EGFR mutation status at diagnosis	
Exon 19 deletion	44 (42.3)
Exon 21 L858R	42 (40.4)
Exon 21 L861Q	2 (1.9)
Exon 18 G719A/G719S/G719X	4 (3.8)
Exon 20 insertion	9 (8.7)
Compound mutation	3 (2.9)
Time from diagnosis advanced to LM (yr)	
≥2	68 (65.4)
<2	36 (34.6)
Brain metastases	
Yes	68 (65.4)
No	36 (34.6)
Extra-CNS metastases	
Yes	73 (70.2)
No	31 (29.8)
CSF cytology	
Positive	92 (88.5)
Negative	12 (11.5)
MRI	
Positive	82 (78.8)
Negative	22 (21.2)
TKIs therapy before LM	
First generation	66 (63.5)
Second generation	9 (8.6)
Third generation	16 (15.4)
Others	3 (2.9)
No	10 (9.6)
TKIs therapy after LM	
First generation	0 (0.0)
Second generation	4 (3.8)
Third generation	95 (91.4)
Others	5 (4.8)
Chemotherapy after LM	
Yes	26 (25.0)
No	78 (75.0)
Bevacizumab after LM	
Yes	44 (42.3)
No	60 (57.7)
Radiotherapy for CNS after LM	
Yes	6 (5.8)
No	98 (94.2)
Ommaya reservoir	
Yes	29 (27.9)
No	75 (72.1)

NSCLC: non-small cell lung cancer; LM: leptomeningeal metastases; KPS: Karnofsky performance status; EGFR: epidermal growth factor receptor; CNS: central nervous system; CSF: cerebrospinal fluid; MRI: magnetic resonance imaging; TKIs: tyrosine kinase inhibitors.

入组的*EGFR*阳性NSCLC-LM患者脑膜转移的临床表现见表2。67.3%患者表现为头痛，34.6%患者表现为恶心和/或呕吐，34.6%患者表现为头晕。18例（17.3%）患者有视神经累及症状，如复视、视物模糊。5例（4.8%）患者有听神经累及症状，如听力下降、耳鸣。8例（7.7%）患者以行走不稳为主要症状。3例（2.9%）患者表现为咬合有障碍（累及三叉神经）。

**表2:** NSCLC-LM患者临床症状（*n*=104）

Clinical symptoms	*n* (%)
Headache	70 (67.3)
Dizziness	36 (34.6)
Nausea and/or vomiting	36 (34.6)
Expression disorder	7 (6.7)
Epilepsy or convulsion	8 (7.7)
Optic nerve involvement	18 (17.3)
Auditory nerve involvement	5 (4.8)
Cognitive impairment	3 (2.9)
Walking instability	8 (7.7)
Masseter muscle dysfunction	3 (2.9)

### 2.2 疗效评估

#### 2.2.1 临床有效性评估

LM患者临床有效性评估见表3。39.4%患者经IP治疗后一般情况改善，KPS评分提高。70例头痛患者中，有65例患者在接受IP治疗后头痛减轻，表现为头痛评分改善。84.6%的患者鞘注后神经系统症状得到了改善。经影像学评估，18例（17.3%）患者脑膜病灶有缓解，66例（63.5%）脑膜病灶稳定，9例（8.6%）患者脑膜病灶进展，11例（10.6%）患者因一般情况较差未能接受影像学评估。69例（66.3%）LM患者脑脊液中CEA指标升高，其中61例（88.4%）患者接受鞘注化疗及靶向药物调整后脑脊液CEA指标有下降。所有患者均纳入总体有效性评估，其中81例（77.9%）患者经IP治疗后评估缓解，14例（13.4%）患者病情稳定，9例（8.7%）患者疾病进展。

**表3:** IP治疗后临床疗效评估

Clinical response	*n*/N (%)
KPS elevation	41/104 (39.4)
Headache score improved	65/70 (92.9)
Neurological symptom remission	88/104 (84.6)
Radiologic evaluation	
Improved	18/104 (17.3)
Stable	66/104 (63.5)
Worse	9/104 (8.6)
Not reviewed	11/104 (10.6)
CSF CEA reduction	61/69 (88.4)
Overall response assessment	
Response	81/104 (77.9)
Stable	14/104 (13.4)
Progression	9/104 (8.7)

IP: intrathecal Pemetrexed; CEA: carcinoembryonic antigen.

#### 2.2.2 生存分析

截止到随访时间，104例患者中有84例达到临床结局（死亡），有20例存活。104例*EGFR*阳性NSCLC-LM患者的中位OS为13.0个月，中位PFS为9.6个月（图1）。6个月OS率为80.8%，1、2、3年OS率分别为56.5%、24.9%和13.3%。亚组分析中，KPS评分高（≥60分）的患者中位OS显著高于KPS评分低（<60分）的患者（14.4 *vs* 9.0个月，*P*=0.0022）（图2A）。规律接受贝伐珠单抗治疗的LM患者中位OS明显高于未接受贝伐珠单抗者（19.2 *vs* 10.5个月，*P*=0.0011，图2C）。考虑到KPS评分、基因突变类型、放化疗等治疗方式对结局的可能影响，为控制混杂因素，本研究进一步采用多变量*Cox*模型校正KPS评分、基因突变类型、治疗方式等变量验证结果的稳定性。结果显示，校正混杂因素后，KPS≥60分患者的死亡风险显著低于KPS<60分患者[风险比（hazard ratio, HR）=0.494，95%CI: 0.314-0.776，*P*=0.002]（图2B），使用贝伐珠单抗组死亡风险显著低于未使用组（HR=0.443, 95%CI: 0.272-0.723, *P*=0.001）（图2D）。*EGFR*经典突变 *vs* 罕见突变、有脑转移 *vs* 无脑转移、有颅外转移 *vs* 无颅外转移、有Ommaya囊 *vs* 无Ommaya囊、接受全身化疗 *vs* 未接受全身化疗、接受头颅放疗*vs*未接受头颅放疗组OS均无统计学差异（图3）。

**图1:**
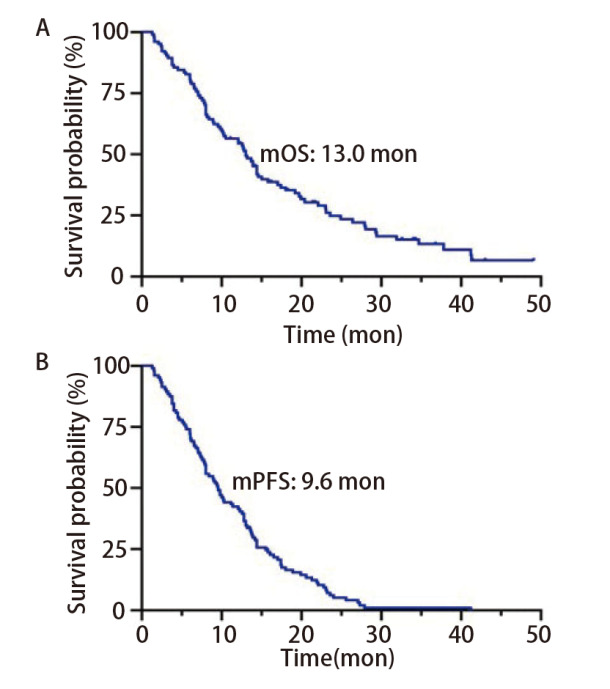
104例NSCLC-LM患者的OS（A）和PFS（B）曲线

**图2:**
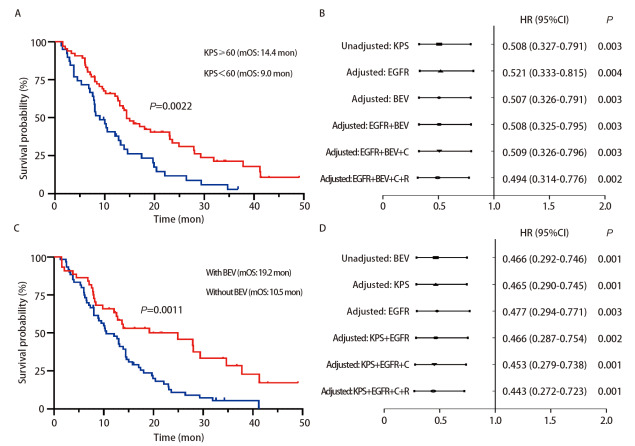
NSCLC-LM患者OS的*Kaplan-Meier*生存分析。 A：KPS≥60分和KPS<60分组的生存曲线；B：调整混杂因素前后，KPS≥60分 *vs* KPS<60分组的死亡风险比；C：贝伐珠单抗组和未使用贝伐珠单抗组的生存曲线；D：调整混杂因素前后，使用贝伐珠单抗 *vs* 未使用贝伐珠单抗组的死亡风险比。

**图3:**
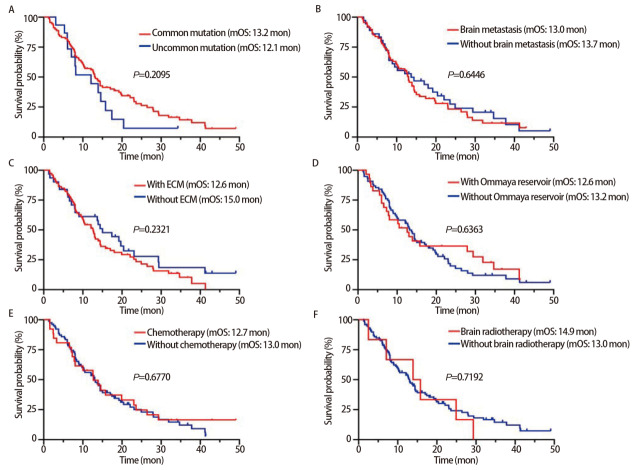
NSCLC-LM患者OS的*Kaplan-Meier*生存分析。 A：经典*EGFR*突变和非经典EGFR突变组的生存曲线；B：合并脑实质转移和不合并脑实质转移组的生存曲线；C：合并颅外转移和不合并颅外转移组的生存曲线；D：有Ommaya囊和无Ommaya囊组的生存曲线；E：接受全身化疗和未接受全身化疗组的生存曲线；F：接受头颅放疗和未接受头颅放疗组的生存曲线。

### 2.3 安全性评估

进一步统计了接受IP治疗患者的AEs，主要包括骨髓抑制（如白细胞减少、血小板减少）、EHA、癫痫发作、急性脑膜炎和脑白质病。骨髓抑制是IP最常见的AEs（表4），占比为58.7%（61例），其中有1例患者鞘注过程中同时出现3级白细胞减少和血小板减少，1例同时出现4级白细胞减少和血小板减少。26例患者出现转氨酶升高，占比25.0%。但大部分AEs属1-2级。有9例患者发生了4级骨髓抑制（占比8.7%），给予对症治疗后均恢复正常。有2例患者鞘注治疗后出现癫痫发作，抗癫痫治疗后好转。无患者发生致命性AEs，如急性脑膜炎和脑白质病。

**表4:** IP治疗后不良反应评估（*n*=104）

Toxicity	n	Grade 1	Grade 2	Grade 3	Grade 4
Myelosuppression	61	7	25	20	9
Leukopenia	53	6	22	18	7
Thrombocytopenia	10	1	3	3	3
EHA	26	23	2	1	0
Epileptic seizure	2	2	0	0	0
Acute cerebral meningitis	0	0	0	0	0
Leukoencephalopathy	0	0	0	0	0

EHA: elevation of hepatic aminotransferase.

## 3 讨论

由于EGFR-TKIs的广泛应用，晚期NSCLC患者生存期明显延长，LM的发生率也随之逐年增加^[3]^。本研究系统地回顾分析了IP治疗在*EGFR*阳性NSCLC-LM患者中的疗效和安全性。结果显示，104例*EGFR*阳性NSCLC-LM患者的中位OS为13.0个月，未观察到严重的中枢神经系统毒性以及治疗相关死亡，提示LM患者可从IP治疗中获益，且AEs可控。

既往多项研究^[8,9]^证实了鞘内注射化疗在NSCLC-LM领域的良好安全性和巨大治疗潜力。临床上常用的鞘内药物包括甲氨蝶呤、阿糖胞苷、塞替派，但主要用于淋巴瘤和白血病患者，在肺癌中应用有限，且副作用较大^[2,14]^。多项研究^[15-17]^结果显示，鞘内注射甲氨蝶呤、阿糖胞苷或塞替派化疗的NSCLC-LM患者的中位OS只有3-6个月。Zhou等^[18]^报道的一项回顾性研究显示，鞘内注射甲氨蝶呤联合全身治疗的NSCLC-LM患者中位OS为10.1个月，但研究仅纳入了7例患者，多数患者接受的是IP治疗。与甲氨蝶呤相似，培美曲塞可通过抑制叶酸代谢阻止肿瘤细胞DNA和RNA合成，进而抑制肿瘤细胞增殖而发挥抗肿瘤作用^[19]^。动物实验^[20]^表明，鞘注的培美曲塞在大鼠脑脊液中能够长时间保持高浓度，提示鞘注培美曲塞作用持久，有利于持久的临床症状改善。本研究LM患者中，颅内压增高引起的头痛、恶心和/或呕吐是最常见的临床表现，严重影响患者的生存质量。IP治疗后，84.6%的患者神经系统症状改善，39.4%的患者KPS评分提高，一般情况好转，临床有效率为77.9%，中位OS超过1年。这与既往一项II期临床试验^[9]^结果相似，IP在NSCLC-LM患者中的临床有效率为80.3%，患者的中位OS为12.0个月。Geng等^[21]^进行的一项回顾性研究结果显示，34例LM患者经IP治疗后的临床有效率为76.5%，中位OS为20个月。但该研究中OS定义与本文不同，OS为确诊LM至死亡或随访时间，如按本文定义从首次IP治疗计算的OS为3.5个月。此外，该研究包含了间变性淋巴瘤激酶（anaplastic lymphoma kinase, *ALK*）突变患者，患者的治疗史不同，且此研究样本量小，这些因素均可能影响OS导致差异化的研究结果。Li等^[12]^和Zhou等^[18]^研究发现经IP治疗的NSCLC-LM患者的中位OS分别为12.0和9.6个月，与本研究结论相一致。因此，IP治疗对*EGFR*阳性NSCLC-LM患者具有可靠的抗肿瘤的疗效，可显著延长患者OS。

亚组分析中，一般状况良好（KPS≥60分）和接受贝伐珠单抗治疗的患者OS更长。KPS评分是评估肿瘤患者身体状况和功能状态的重要工具。Yin研究团队^[22]^建立了一个整合分子信息的肺癌脑膜转移预后模型，其中KPS评分被证实是独立预后影响因素。KPS评分与治疗的耐受性和有效性密切关联。KPS评分较高的患者更有可能完成全程治疗，接受多次鞘内注射化疗，并从靶向治疗、化疗、放疗等各种治疗手段中获益。此外，本研究发现贝伐珠单抗治疗可改善*EGFR*阳性NSCLC-LM患者的预后，进一步延长患者OS。贝伐珠单抗是一种重组人源化单克隆抗体，通过特异性结合血管内皮生长因子（vascular endothelial growth factor, VEGF），阻断其与受体的结合，抑制肿瘤血管生成，进而发挥抗肿瘤作用^[23]^。Yi等^[24]^报道的一项回顾性研究结果表明，贝伐珠单抗联合奥希替尼治疗*EGFR*突变NSCLC-LM患者的生存期为18.0个月，明显长于只接受奥希替尼治疗组的13.7个月。其机制可能是贝伐珠单抗抑制不成熟的血管生成，诱导血管正常化，从而在联合治疗中增加奥希替尼的颅内浓度，调节钙黏蛋白水平和下调*EGFR*和下游信号通路，进而发挥抗肿瘤作用。另有研究^[23,25,26]^显示，抗血管生成治疗可通过抑制血管生成，减少脑转移患者的脑水肿和颅内压升高，改善BBB通透性，增强联合治疗的入脑疗效，进而改善患者的症状和生活质量，延长患者的OS。因此，贝伐珠单抗可能通过阻断VEGF信号诱导血管正常化，降低软脑膜间质压并改善局部灌注，进而提升培美曲塞在脑脊液中的分布均匀性及肿瘤组织渗透浓度，强化其细胞毒效应^[23]^。此外，贝伐珠单抗还可显著提高EGFR*-*TKIs在颅内病灶的药物暴露量，进一步放大二者协同抗肿瘤作用^[24]^，最终在LM的控制中发挥叠加优势。未来仍需相关研究进一步探索贝伐珠单抗与其他治疗手段（如免疫治疗）的联合应用，以优化治疗方案，提高患者的预后。

本研究中接受全脑放疗后，LM患者的OS并不能得到改善，这可能与照射范围不足、剂量低以及广泛的AEs有关。此外，置入Ommaya囊进行IP治疗组患者的OS与经腰穿IP治疗组相仿，差异无统计学意义，这与Delgado团队^[27]^的研究结果相反。该研究纳入30例经腰椎穿刺鞘内化疗和10例经Ommaya囊鞘内化疗的脑膜转移患者，结果显示经Ommaya鞘内化疗组OS显著高于腰穿组（分别为9.2和4个月），证实了Ommaya囊对脑膜转移患者的生存获益。但是此研究纳入对象是多种恶性肿瘤患者，如乳腺癌、肺癌、卵巢癌、白血病等，其中肺癌患者只有7例。这种结果的差异可能与入组患者的基线特征、治疗手段等有关。

本研究中纳入的患者均携带*EGFR*突变，且所有*EGFR*经典突变患者均同时使用第三代TKIs靶向治疗作为全身治疗。研究^[5,6,28]^显示，第三代EGFR-TKIs（奥希替尼、伏美替尼、阿美替尼）具有更强的BBB穿透能力，从而能够更有效地控制颅内病灶，改善合并脑实质和脑膜转移转移患者的预后。但在LM的靶向治疗中，由于BBB的存在，脑脊液中的EGFR-TKIs浓度通常不足。BLOOM研究^[28]^结果显示，每天口服奥希替尼160 mg可显著改善*EGFR*突变NSCLC-LM患者的生存期和神经功能，中位PFS为8.6个月，中位OS突破11个月，远高于传统治疗。因此，为了克服这一缺陷，根据BLOOM研究结果，本研究团队增加了口服EGFR-TKIs的剂量，本研究中所有经典突变患者均接受双倍剂量第三代EGFR-TKIs治疗，TKIs联合IP治疗后患者的OS可达13.0个月。一项前瞻性、多中心队列研究^[29]^也证实了双倍奥希替尼在NSCLC-LM患者中的疗效和安全性。该研究共纳入40例*EGFR* T790阳性的LM患者，所有患者均接受160 mg奥希替尼靶向治疗，颅内疾病控制率（disease control rate, DCR）达到92.5%，中位OS可达13.3个月（95%CI：9.1-未达到），且AEs可控，大多数AEs是1-2级。Chen等^[30]^进行的一项前瞻性研究结果显示，高剂量伏美替尼（240 mg/d）显示出良好的疗效和安全性，中位OS为8.43个月，临床有效率为75%。鞘内化疗联合双倍剂量TKIs治疗可协同增强BBB穿透性，进而改善LM患者的生存，但其广泛应用仍需更多循证医学证据支持。

与既往多项研究^[9,21]^结果相似，骨髓抑制和转氨酶升高是鞘注化疗常见的AEs。本研究中骨髓抑制和转氨酶升高发生率略高，其可能与多次鞘注化疗药物累积和既往联合全身治疗有关，但大多数为1-2级，AEs可控，未观察到急性脑膜炎或脑白质病等严重AEs。这些结果表明，IP治疗在NSCLC伴LM患者中具有良好的耐受性，且AEs可控。

本研究存在一些局限性，作为一个回顾性研究，可能存在选择偏倚和混杂因素。另外，部分患者接受了多种联合治疗（如靶向治疗、抗血管生成治疗、放疗等），抗肿瘤方案的异质性可能影响对单一治疗效果的评估。期待后续更大规模的前瞻性研究来进一步探索鞘注化疗的疗效和安全性。

在*EGFR*阳性NSCLC伴LM患者中，IP可有效控制疾病进展、减缓症状和延长OS，同时具有良好的安全性和耐受性，联合使用贝伐珠单抗或双倍剂量的第三代EGFR-TKIs可进一步改善预后。
